# Adipose‐Derived Stem Cell Exosomes in Diabetic Wound Repair: Molecular Crosstalk, Bioengineering Strategies, and Translational Challenges

**DOI:** 10.1155/sci/4151678

**Published:** 2026-09-18

**Authors:** Danna Yao, Yujie Xiao, Mali Zhou, Panpan Sun, Ling Wang, Hongtao Wang

**Affiliations:** ^1^ School of Medicine, Northwest University, Xi’an 710069, Shaanxi, China, nwu.edu.cn; ^2^ Department of Burns and Cutaneous Surgery, Xijing Hospital, Fourth Military Medical University, Xi’an 710032, Shaanxi, China, fmmu.edu.cn

**Keywords:** adipose-derived stem cell exosomes, diabetic wounds, engineered exosomes, intelligent delivery

## Abstract

Diabetic wounds are sustained by persistent inflammation, impaired angiogenesis, abnormal extracellular matrix (ECM) remodeling, and delayed re‐epithelialization. Adipose‐derived stem cell exosomes (ADSC‐Exos) offer a cell‐free strategy because their multicomponent cargo can modulate immune responses, vascular regeneration, fibroblast activity, and epidermal repair. Mechanistic studies suggest that ADSC‐Exos promote macrophage immunometabolic reprogramming, restore pro‐angiogenic signaling, improve matrix homeostasis, and protect keratinocytes from oxidative injury. Engineering approaches and biomaterial‐based delivery systems may further improve local retention, controlled release, and therapeutic activity. Distinct from existing ADSC‐Exos reviews, this review adopts a pathology‐to‐translation framework that connects the major pathological features of diabetic wounds with ADSC‐Exos mechanisms, engineering strategies, delivery optimization, and clinical translation. Particular emphasis is placed on donor‐ and process‐dependent heterogeneity, potency assessment, GMP‐compatible manufacturing, biodistribution, long‐term safety, and regulation. Overall, ADSC‐Exos represent a mechanistically compelling preclinical platform, but clinical translation will require reproducible product quality, standardized potency criteria, scalable manufacturing, and rigorous clinical validation.

## 1. Introduction

Diabetes mellitus is a metabolic disorder caused by the combined effects of genetic and environmental factors. Sustained hyperglycemia can lead to secondary damage involving multiple systems and organs, including diabetic nephropathy, retinopathy, and neuropathy [1–5]. Among these complications, chronic diabetic wounds are some of the most serious and difficult to treat [6]. According to 2023 statistics from the International Diabetes Federation, ~15%–25% of patients with diabetes worldwide will develop chronic wounds during the course of the disease. More than 1 million amputations are performed each year due to diabetic foot ulcers (DFUs), and the 1‐year mortality rate after amputation exceeds 20% [7]. Current clinical management mainly relies on multimodal interventions, including debridement, infection control, offloading, negative pressure wound therapy, and topical application of growth factors [8–11]. However, the overall healing rate remains unsatisfactory [12–14].

In recent years, mesenchymal stem cells (MSCs) have been widely investigated for wound therapy because of their paracrine‐mediated effects on tissue repair [15, 16]. Adipose‐derived stem cells (ADSCs) have become one of the most commonly used stem cell types for potential clinical application, owing to their easy accessibility, abundant tissue source, and minimally invasive harvest procedure [17, 18]. However, the clinical use of living cell‐based therapies is still challenged by several concerns, including immune rejection, tumorigenic risk, and difficulties in storage and transportation. Increasing evidence indicates that the tissue‐reparative effects of ADSCs are mainly mediated by their secreted exosomes [19]. ADSC‐Exos contain diverse bioactive molecules, including proteins, lipids, and nucleic acids, which can be taken up by recipient cells through endocytosis, membrane fusion, and receptor‐ligand interactions, thereby regulating intracellular signaling pathways and gene expression in target cells and contributing to tissue repair [20–22]. Compared with living cell transplantation, ADSC‐Exos may offer relative safety and logistical advantages as cell‐free products; however, these advantages are conditional rather than standardized, because donor and process variability can alter product composition and potency [23]. Clinical translation therefore still requires harmonized manufacturing and quality‐control standards, as well as improved persistence and targeting. For engineered products, clinical translation additionally requires control of stability, biodistribution, impurities, and regulatory complexity [24, 25]. This review focuses on the pathological features of the diabetic wound microenvironment and systematically summarizes the mechanisms by which ADSC‐Exos promote diabetic wound repair. We further discuss the major barriers to clinical translation, including donor heterogeneity, standardized preparation, quality control, safety evaluation, and regulatory considerations. In addition, engineering‐based enhancement strategies, optimization of delivery systems, and future translational prospects are reviewed, with the aim of providing a reference for basic research and clinical application of ADSC‐Exos in the treatment of chronic diabetic wounds.

## 2. Pathologic Microenvironment Underlying Impaired Diabetic Wound Healing

Normal wound healing is a highly coordinated process that involves four overlapping phases: hemostasis, inflammation, proliferation, and remodeling [26]. However, in the diabetic setting, sustained hyperglycemia reshapes the local wound microenvironment through metabolic dysregulation, vascular dysfunction, and immune imbalance. As a result, the wound remains locked in a prolonged inflammatory state and ultimately progresses into a chronic nonhealing wound [27–29].

### 2.1. Persistent Inflammation and Immune Imbalance

The main function of early inflammatory response is to eliminate pathogens and necrotic tissue, after which inflammation is resolved and immune remodeling supports the transition toward tissue regeneration. Under persistent hyperglycemic stimulation, however, glucose metabolism is disrupted, and diabetic wounds show typical features of excessive inflammatory activation and delayed resolution. High glucose levels and the accumulation of advanced glycation end products (AGEs) activate inflammatory pathways, including NF‐κB and the NLRP3 inflammasome, leading to sustained release of pro‐inflammatory cytokines, such as TNF‐α, IL‐1β, and IL‐6, as well as excessive production of reactive oxygen species (ROS), which directly impairs the ability of macrophages to polarize toward the reparative M2 phenotype [30]. Among the underlying mechanisms, defective expression of the epigenetic regulator Setdb2 has been identified as a key molecular event that contributes to impaired macrophage phenotype switching in diabetic wounds [31]. Disruption of the IFN*β*‐Setdb2 signaling axis results in persistent activation of inflammatory genes, thereby maintaining macrophages in a pro‐inflammatory M1 state [32, 33]. Continuously activated M1 macrophages secrete large amounts of inflammatory mediators and show abnormally high expression of matrix metalloproteinases (MMPs), particularly MMP‐9 [34, 35]. MMP‐9 can specifically cleave integrin *β*2 on the macrophage surface, further disturbing the normal regulation of macrophage function and leading to sustained and uncontrolled inflammation [36, 37]. On the other hand, persistent neutrophil infiltration and impaired monocyte chemotaxis in diabetic wounds cause severe immune dysfunction, markedly reducing pathogen clearance and allowing recurrent infection to further amplify the inflammatory response [37, 38]. In addition, recent studies have shown that macrophages in diabetic wounds are not only defective in phenotypic transition but also exhibit abnormalities in mitochondrial and lipid metabolism. These changes are characterized by sustained glycolytic activity, suppressed mitochondrial oxidative phosphorylation, and reduced autophagic flux, which together help maintain a chronic pro‐inflammatory state [39].

### 2.2. Impaired Angiogenesis and Tissue Hypoxia

Angiogenesis is a central event in wound repair, as it determines oxygen delivery, nutrient supply, and the recruitment of reparative cells into the injured tissue. Diabetic wounds commonly exhibit endothelial dysfunction, reduced microvascular perfusion, and local tissue hypoxia. On the one hand, inflammatory mediators and MMPs disrupt the pro‐angiogenic wound microenvironment, impair endothelial cell function, and weaken vascular endothelial growth factor (VEGF) signaling, resulting in poor neovascularization [8]. On the other hand, abnormal glucose metabolism and increased local oxygen consumption cause chronic hypoxia in diabetic wounds. Under physiological conditions, this hypoxic stimulus should activate the hypoxia‐inducible factor‐1 *α* (HIF‐1 *α*) pathway and initiate compensatory angiogenesis [40]. However, high glucose and oxidative stress reduce the stability of HIF‐1 *α* protein, leading to subsequent downregulation of target genes, including VEGF. As a consequence, endothelial cell proliferation, migration, and new capillary formation are suppressed [41]. In addition, the hemostatic function of platelets and coagulation factors is impaired in a high‐glucose environment, which is mainly reflected by reduced clot stability [42]. Because the blood clot serves as an important provisional matrix for angiogenesis within the wound bed, reduced clot stability can hinder endothelial cell migration and capillary sprouting. Meanwhile, hyperglycemia promotes nuclear translocation of YAP in endothelial cells, upregulates COX‐2/mPGES‐1, and increases the production of prostaglandin E_2_ (PGE_2_). PGE_2_ then acts on platelet EP3 receptors, inducing abnormal platelet aggregation and disorganized clot structure [43]. Together with these changes, microvascular lesions and local thrombosis further restrict blood perfusion in the wound. Moreover, the interaction between AGEs and RAGE interferes with the effective release of pro‐repair growth factors, such as platelet‐derived growth factor (PDGF) and transforming growth factor‐*β* (TGF‐*β*), weakens local cell chemotaxis, migration, and angiogenic signaling, thus causing persistent ischemia, hypoxia, and insufficient angiogenesis in diabetic wounds [44].

### 2.3. Abnormal ECM Remodeling and Impaired Fibroblast Function

During wound healing, the extracellular matrix (ECM) not only provides a structural scaffold for cell migration and tissue reconstruction but also participates in signal regulation by storing and releasing multiple cytokines and growth factors. MMPs are key mediators of ECM remodeling through the degradation of collagen, fibronectin, and other ECM components, and their activity is dynamically controlled by tissue inhibitors of metalloproteinases (TIMPs). Therefore, the balance between MMPs and TIMPs is essential for proper ECM remodeling and tissue repair [45]. However, in diabetic wounds, persistent hyperglycemia, oxidative stress, and chronic inflammation jointly lead to excessive MMP expression and relatively insufficient TIMP activity. This imbalance results in excessive ECM degradation, reduced collagen deposition, and disruption of the basement membrane structure [46]. At the same time, the binding of AGEs to RAGE activates inflammatory signaling pathways, such as NF‐κB, promotes abnormal cross‐linking of ECM proteins, and suppresses collagen synthesis. These changes increase ECM stiffness and further interfere with the normal remodeling process [47]. At the cellular level, a high‐glucose microenvironment markedly inhibits fibroblast proliferation, migration, and collagen‐producing capacity, leading to insufficient granulation tissue formation and impaired wound contraction. More importantly, fibroblasts in diabetic wounds may acquire a senescence‐like phenotype, characterized by reduced proliferative potential, an abnormal secretory profile, and a weakened response to reparative signals [48]. This may represent an important cellular basis for the long‐term nonhealing nature of diabetic wounds.

### 2.4. Delayed Re‐Epithelialization and Loss of the Epidermal Barrier

Keratinocyte migration and proliferation are critical for wound closure and restoration of the epidermal barrier. During normal wound repair, keratinocytes at the wound edge gradually detach from the basement membrane under the coordinated regulation of inflammatory mediators, growth factors, and ECM signals. They then migrate toward the wound center and complete epidermal coverage through subsequent proliferation, stratification, and differentiation. However, in the diabetic microenvironment, persistent hyperglycemia, accumulation of AGEs, excessive ROS production, insufficient local growth factor availability, and sustained release of chronic inflammatory mediators collectively impair keratinocyte migration and proliferation. As a result, epithelial tongue advancement from the wound edge is delayed, leading to prolonged wound exposure [49–51]. Recent studies have shown that defective re‐epithelialization in diabetic wounds is not simply caused by reduced keratinocyte function. Instead, it reflects an imbalanced wound microenvironment involving epidermal cells, immune cells, nerve endings, and the ECM. Diabetes‐associated neuropathy can reduce neuropeptide release and weaken neuro‐epidermal trophic support, thereby further affecting keratinocyte activation [52]. In addition, the persistent inflammatory state of diabetic wounds can alter the epidermal stem cell niche, impairing their self‐renewal and differentiation capacity and reducing the regenerative reserve of the epidermis. Long‐term loss of the epidermal barrier also increases the invasion of external pathogens and wound exudation, which further aggravates local inflammation [53]. These findings suggest that future therapeutic strategies should not only promote keratinocyte proliferation and migration but also improve oxidative stress, immune‐inflammatory status, neuro–epidermal interactions, and the ECM microenvironment.

**Figure 1:**
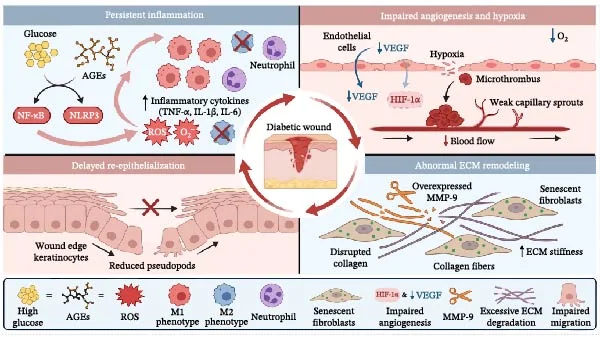
Pathological mechanisms of diabetic wounds. Hyperglycemia‐induced ROS, AGEs, and inflammatory signaling sustain inflammation, while hypoxia, reduced VEGF, and microthrombi impair angiogenesis. Delayed keratinocyte migration and abnormal ECM remodeling further hinder repair, resulting in chronic nonhealing wounds.

Overall, diabetic wounds are characterized by a complex pathological network involving persistent inflammation, immune‐metabolic imbalance, impaired angiogenesis, abnormal ECM remodeling, delayed re‐epithelialization, and increased susceptibility to infection [54–56]. Based on these findings, we propose a schematic summary in Figure 1. These pathological processes do not occur in isolation; rather, they reinforce each other and form a vicious cycle. Therefore, the key to treating diabetic wounds should not be limited to promoting the proliferation of a single cell type or activating an individual signaling pathway. Instead, comprehensive intervention based on coordinated multitarget and multistage regulation is needed. ADSC‐Exos, with their endogenous multicomponent molecular cargo, have potential advantages in regulating multiple signaling pathways, providing an important theoretical basis for their development as candidate therapeutic strategies for diabetic wound treatment.

## 3. Biological Basis, Preparation, and Quality Control of ADSC‐Exos

### 3.1. Nomenclature, Biogenesis, and Compositional Characteristics of ADSC‐Derived EVs

Extracellular vesicles (EVs) are heterogeneous nanoscale vesicles that are actively secreted by cells and enclosed by a lipid bilayer membrane. They are widely present in various body fluids and tissue microenvironments and participate in intercellular material transport and information exchange. EVs play important roles in immune regulation, tissue repair, inflammatory responses, and other biological processes [57]. However, because different studies vary in terms of vesicle isolation methods, particle size ranges, biogenesis pathways, and criteria for marker identification, the terminology used in this field has not yet been fully standardized. Commonly used terms include “EVs,” “exosomes,” “small extracellular vesicles (sEVs),” and “microvesicles (MVs)” [58]. MISEV2023 recommends generic EV terminology and the use of operational terms such as small EVs (sEVs) unless endosomal biogenesis has been directly demonstrated [59]. Because most studies cited here identify ADSC‐derived vesicles by size, morphology, and EV markers rather than direct evidence of biogenesis, we retain “ADSC‐Exos” only as a literature‐consistent shorthand; throughout this review, it should be interpreted as ADSC‐derived sEV‐enriched preparations rather than a biogenesis‐defined exosome population. Canonical exosomes arise as intraluminal vesicles within multivesicular bodies and are released after fusion with the plasma membrane, but the size range and marker expression alone do not establish this origin [59]. These vesicles can carry proteins, lipids, and nucleic acids, such as mRNAs, miRNAs, and long noncoding RNAs (lncRNAs). DNA‐related components have also been detected in some studies. Therefore, ADSC‐Exos serve as important carriers for intercellular communication [60, 61].

### 3.2. Isolation, Purification, and Characterization of ADSC‐Exos

The isolation and purification methods used for ADSC‐Exos directly determine the purity, biological activity, and batch‐to‐batch reproducibility of the final preparation. Variability in these methods is one of the core reasons for the large discrepancies and poor reproducibility observed across studies in this field. Current methods for ADSC‐Exos isolation include ultracentrifugation, density gradient centrifugation, size‐exclusion chromatography (SEC), immunoaffinity capture, and commercial precipitation kits. The basic principles, advantages, and limitations of these methods are summarized in Table 1. Although differential ultracentrifugation (dUC) remains widely used for laboratory‐scale EV enrichment, MISEV2023 notes that high‐speed dUC can co‐isolate nonvesicular particles and promote EV aggregation; recovery is also sensitive to centrifugation parameters [59, 62–64].

**Table 1:** Comparison of the advantages and disadvantages of commonly used EV/sEV separation methods.

Isolation method	Principle	Advantages	Disadvantages	References
Ultracentrifugation	Vesicles are separated according to sedimentation coefficients from large to small particles by gradually increasing centrifugal force, based on differences in particle size and density.	Widely used in laboratory research; broad sample applicability	Limited purity; risk of particle cross‐contamination and vesicle aggregation; low recovery efficiency in protein‐rich samples.	[62–64]
Density gradient centrifugation	Based on buoyant density differences, extracellular vesicles migrate to corresponding density layers during density‐gradient ultracentrifugation.	Enables effective separation of different vesicle subtypes according to density; reduces protein contamination; cushion‐based approaches are relatively simple to perform.	Time‐consuming; requires subsequent removal of density‐gradient media; overall recovery rate is relatively low.	[65, 66]
Size‐exclusion chromatography	Based on particle size differences, separation is achieved using a porous matrix column, in which larger particles elute earlier and smaller particles elute later.	Gentle and simple operation; column materials can be reused.	Samples are diluted and require subsequent concentration; separation efficiency is affected by multiple parameters; potential cross‐contamination may occur.	[67, 68]
Microfluidics‐based method	Based on hydrodynamic properties, such as size and isoelectric point, particles are separated in a flow system without a stationary phase.	Suitable for heterogeneous samples; high recovery rate; gentle operation that preserves the native state of particles.	Requires specialized equipment and parameter optimization to achieve optimal separation performance.	[69–71]
Immunoaffinity chromatography	Based on specific molecular markers, target vesicles are captured through affinity probes, such as antibodies, peptides, or lectins, immobilized on a matrix.	Highly specific isolation of particular extracellular vesicle subpopulations; suitable for functional studies and precise sorting.	Nonspecific binding may occur; elution can be difficult and may damage EV structure; recovery is limited and probe specificity must be rigorously validated.	[72]
Commercial kit‐based extraction	Combination of multiple isolation techniques; EVs are sequentially separated according to different physicochemical properties to improve specificity.	High purity and specificity; enables efficient recovery from conditioned media.	Core reagent components and purification mechanisms are usually not fully disclosed; risk of contaminants and difficulty in interpreting results.	[59, 62]

Commonly used methods for exosome characterization include transmission electron microscopy or cryo‐electron microscopy to observe vesicle morphology; nanoparticle tracking analysis to determine particle size and concentration [73], and Western blotting to detect marker proteins such as CD9, CD63, CD81, TSG101, and Alix [74]. However, the selection of positive marker combinations varies among different studies. For example, some studies detect only CD63 and CD81 while lacking validation of CD9 or TSG101. The reported particle‐size distribution ranges are also inconsistent. Moreover, most preclinical studies do not adequately evaluate preparation purity, which may affect the accuracy and reproducibility of dose calculations in subsequent functional experiments. Therefore, according to the updated MISEV2023 recommendations issued by the ISEV, exosome studies should, whenever possible, report positive marker proteins, markers of nonvesicular contaminants, particle size distributions, particle numbers, and preparation sources simultaneously to improve the reliability of the results [59, 75].

### 3.3. Source Advantages and Therapeutic Potential of ADSC‐Exos

ADSCs are widely available, relatively easy to obtain, associated with minimal harvest‐related injury, and capable of yielding large numbers of cells, making them particularly suitable for large‐scale exosome production [76, 77]. Compared with BMSC‐Exos, ADSC‐Exos offer greater accessibility and higher cellular expansion efficiency; compared with HUCMSC‐Exos, ADSC‐Exos are associated with a lower ethical burden. A detailed comparison of these characteristics is presented in Table 2. Although exosomes from different sources share similar surface markers and functions, direct comparative studies of exosomes derived from different MSC sources in diabetic wounds are limited. Therefore, it cannot yet be concluded that ADSC‐Exos are superior to exosomes from other MSC sources in all chronic wound scenarios. Future studies should perform head‐to‐head comparisons under standardized conditions to clarify the optimal application contexts for exosomes derived from different cellular sources.

**Table 2:** Comparison of the characteristics of ADSCs, ADSC‐Exos, and exosomes derived from other stem cell sources.

Type	Source acquisition	Size (nm)	Markers (partial)	Functional tendency	Limitations	References
ADSC‐Exos	Byproducts of liposuction; minimally invasive	30–150	CD63^+^, CD81^+^, CD9^+^, TSG101	Pro‐angiogenic, anti‐inflammatory, anti‐fibrotic	Need for standardized purification criteria; short in vivo half‐life	[78–80]
BMSC‐Exos	Bone marrow aspiration; invasive	40–160	CD63^+^, CD81^+^, CD9^+^, Integrins	Osteogenic/chondrogenic differentiation, immunosuppression, treatment of hematologic disorders	Greater donor‐site trauma, faster cellular senescence, limited yield	[81, 82]
HUCMSC‐Exos	Umbilical cord	30–150	CD63^+^, CD81^+^, CD9^+^, HLA‐G	Osteogenesis, immunosuppression, treatment of hematologic disorders	Ethical approval requirements, batch‐to‐batch variability	[83, 84]

As key mediators of the paracrine effects of their parental cells, ADSC‐Exos carry multiple classes of nucleic acids, including mRNAs, miRNAs, lncRNAs, and certain DNA‐related molecules [85]. They can efficiently transfer bioactive molecules and are more amenable than living cells to engineering and controlled handling, with relatively low immunogenicity and favorable safety profiles reported in preclinical studies [86–88]. Studies have shown that ADSC‐Exos can cross multiple biological barriers and be taken up by macrophages, endothelial cells, fibroblasts, keratinocytes, and other cell types, thereby regulating inflammatory responses, metabolic status, migration, proliferation, and differentiation. For example, Shin et al. [89] analyzed the protein and lipid components of ADSC‐Exos, investigated their role in skin barrier repair, and reported that ADSC‐Exos enhanced stratum corneum hydration. In addition, ADSC‐Exos can be absorbed and internalized by fibroblasts, stimulating cell migration, proliferation, and collagen synthesis, while increasing the gene expression of N‐cadherin, cyclin‐1, proliferating cell nuclear antigen, and collagen types I and III [90]. They can also promote vascular endothelial cell proliferation, migration, and tube formation by delivering various cytokines and miRNAs, while reducing macrophage infiltration and the secretion of proinflammatory cytokines [78]. Together, these studies support the mechanistic potential of ADSC‐Exos to modulate multiple wound‐repair pathways, but this activity should not be considered a batch‐invariant property. Donor traits, culture microenvironments, and isolation methods can substantially alter cargo composition and thereby functional potency [91]. Reproducible multitarget efficacy will therefore require standardized manufacturing and potency‐based batch qualification.

## 4. Mechanisms by Which ADSC‐Exos Promote Diabetic Wound Repair

**Figure 2:**
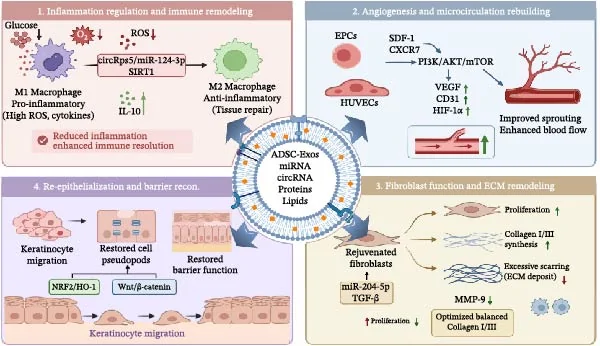
ADSC‐Exos in diabetic wound healing. ADSC‐Exos functional cargo to wound cells, reducing inflammation, promoting angiogenesis, improving fibroblast‐mediated ECM remodeling, and enhancing keratinocyte migration and barrier repair, thereby accelerating diabetic wound healing.

ADSC‐Exos have become an important focus of translational research in wound repair and skin regeneration. Current evidence indicates that ADSC‐Exos exert multiple regulatory effects during diabetic wound healing (Figure 2), and their mechanisms involve complex molecular regulatory networks rather than a single signaling pathway. Notably, most mechanistic studies to date have been based on in vitro high‐glucose models and rodent wound models. In addition, there are considerable differences among studies in the source of ADSCs, methods used for exosome isolation, administration dose, and outcome assessment. Accordingly, current findings support mechanistic plausibility and qualitative therapeutic trends, but heterogeneity in experimental design limits quantitative cross‐study comparison and precludes a unified dose–response or efficacy model. In the following sections, the effects of ADSC‐Exos are integrated and discussed according to several key pathological processes involved in diabetic wound healing.

### 4.1. Regulation of Inflammatory Responses and Immune Remodeling

ADSC‐Exos have been shown to markedly reduce excessive intracellular ROS production under high‐glucose conditions, downregulate the expression of inflammatory cytokines such as IL‐1β, IL‐6, and TNF‐α, and activate endogenous antioxidant pathways. Through these effects, ADSC‐Exos can alleviate high glucose‐induced epidermal cell injury and local inflammatory responses [92]. In parallel, ADSC‐Exos promote the expression of anti‐inflammatory mediators, including IL‐10, prostaglandin E2 (PGE2), and indoleamine 2,3‐dioxygenase (IDO), suppress excessive T lymphocyte proliferation, and induce the expansion of regulatory B cells. These regulatory effects help maintain peripheral tolerance and support the transition of wounds from the inflammatory phase to the proliferative phase [93].

Regulation of macrophage polarization is a central component of immune remodeling during wound repair [94]. In diabetic wounds, the persistence of M1 macrophages and impaired transition toward the M2 phenotype prolong inflammation and further inhibit angiogenesis and ECM deposition [95–97]. *CircRps5*, a key circular noncoding RNA carried by ADSC‐Exos, has been reported to promote macrophage polarization toward the M2 phenotype by regulating miR‐124‐3p expression, thereby correcting the local imbalance of macrophage phenotypes in the wound microenvironment [98]. In addition, ADSC‐Exos can modulate the expression of monocyte chemoattractant protein‐1 (MCP‐1), macrophage colony‐stimulating factor (M‐CSF), and granulocyte‐macrophage colony‐stimulating factor (GM‐CSF), which further facilitates M2 macrophage polarization [99]. However, optimal repair of diabetic wounds does not rely simply on broad suppression of inflammation. Instead, it requires restoration of the proper temporal pattern of the inflammatory response, allowing inflammation to be initiated in a timely manner, pathogens and debris to be effectively cleared, and the inflammatory response to resolve in an orderly way before transition to tissue repair. Therefore, future studies should not only assess inflammatory cytokine levels and M1/M2 polarization markers, but also determine whether ADSC‐Exos can promote neutrophil clearance, drive reparative macrophage reprogramming, and reduce tissue damage associated with chronic inflammation.

Recent studies further suggest that the effects of ADSC‐Exos on macrophages are not limited to phenotypic switching, but also involve immunometabolic reprogramming. Specifically, ADSC‐Exos may help rebuild a reparative immune phenotype by restoring mitochondrial homeostasis, autophagy, and lipid metabolism. Bai et al. [100] showed that ADSC‐Exos improved mitochondrial function and restored autophagic flux in macrophages through activation of SIRT1‐related pathways, thereby reversing persistent M1 polarization. These findings indicate that immunometabolic reprogramming may represent an important mechanism by which ADSC‐Exos alleviate chronic inflammation and promote immune remodeling in diabetic wounds.

Nevertheless, the M1/M2 interpretation requires caution. Most ADSC‐Exos studies associate efficacy with increased M2 markers, whereas single‐cell analysis of human DFUs found more M1 macrophages in healers and more M2 macrophages in nonhealers [101]. Single‐cell profiling of diabetic mouse wounds further identified multiple macrophage subsets with distinct transcriptional programs [102]. These findings indicate that “more M2” does not necessarily predict better healing and that the classical M1/M2 framework may oversimplify macrophage heterogeneity. Thus, ADSC‐Exos may act by restoring the temporal and functional balance of macrophage responses rather than simply driving sustained M2 polarization; future studies should incorporate longitudinal and single‐cell analyses.

### 4.2. Promotion of Angiogenesis and Microcirculatory Reconstruction

Endothelial progenitor cells (EPCs), a population of immature endothelial cells with strong migratory capacity, play an important role in neovascularization of ischemic tissues and repair of vascular injury. Previous studies have shown that high‐glucose conditions can induce premature senescence of EPCs, leading to ROS accumulation and aggravated inflammatory responses. Li et al. [103] reported that ADSC‐Exos exert multiple protective effects on EPCs through activation of the stromal cell‐derived factor‐1 (SDF‐1)/C‐X‐C chemokine receptor type 7 (CXCR7) signaling axis. These effects include increased secretion of VEGF, enhanced phosphorylation of VEGF receptor 2 (VEGFR2), and reduced ROS production and inflammatory cytokine release.

In addition, ADSC‐Exos can promote neovascularization by activating a series of signaling pathways that enhance endothelial cell proliferation, migration, and tube formation. They also increase the expression of angiogenesis‐related molecules, including VEGF, CD31, CD34, and HIF‐1α. For example, Che et al. [104] performed miRNA‐seq analysis and found that miR‐146a‐5p, which is highly enriched in ADSC‐Exos, targets and regulates the JAZF1 gene. This activates downstream ERK/NF‐κB signaling and subsequently upregulates VEGFA expression in endothelial cells. Moreover, hypoxia‐preconditioned ADSC‐Exos contain high levels of miR‐100‐5p, which enhances the proliferation, migration, and tube‐forming ability of human umbilical vein endothelial cells (HUVECs) in vitro by regulating signaling pathways such as PI3K/AKT/mTOR. In a rat model of DFU, these exosomes effectively reduced ulcer area and increased VEGF/CD31 expression [105]. However, markers such as VEGF and CD31 mainly reflect early angiogenic activity and are not sufficient to demonstrate the formation of a structurally stable, well‐perfused, and long‐lasting mature vascular network. In diabetic wounds, high‐quality vascular reconstruction also depends on vascular smooth muscle cell coverage, basement membrane stability, and restoration of local perfusion. Future studies should therefore include vascular maturation markers such as *α*‐smooth muscle actin (α‐SMA) and NG2, laser Doppler blood flow analysis, and assessment of tissue hypoxia to further verify whether ADSC‐Exos can promote mature vessel formation and improve local microcirculation in diabetic wounds.

HIF‐1α is a key transcriptional regulator of VEGF, but its activity is often suppressed under high‐glucose conditions. By delivering functional miRNAs and proteins, ADSC‐Exos can restore HIF‐1α signaling activity and thereby upregulate the expression of pro‐angiogenic factors such as VEGF [106]. After injection of miR‐125b‐modified ADSC‐Exos in a DFU rat model, the expression of VEGF and TGFβ‐1 in wound tissues was significantly increased, whereas the expression of the angiogenesis inhibitor DLL‐4 was effectively suppressed, together promoting neovascularization [107]. In addition, engineered ADSC‐Exos overexpressing Tβ4 can increase angiogenic capacity through the activation of the PI3K‐AKT‐mTOR/HIF‐1α signaling axis [108]. Recent studies have further amplified the pro‐angiogenic effects of engineered exosomes by combining them with biomaterials. For instance, a HAMA‐PLMA two‐photon polymerized hydrogel loaded with Tβ4‐Exos provided sustained pro‐angiogenic signals at diabetic wound sites through controlled release and upregulation of HIF‐1α signaling. This strategy enabled coordinated regulation of angiogenesis and macrophage polarization during diabetic wound repair [109].

Although current studies consistently report pro‐angiogenic effects of ADSC‐Exos, their mechanistic conclusions are not fully comparable because different studies use different cargo, pathways, doses, and mainly surrogate endpoints such as VEGF or CD31. Importantly, diabetic wounds are characterized not only by reduced vessel formation but also by increased vascular permeability and deficient pericyte coverage [110]. Therefore, HIF‐1α/VEGF‐centered evidence should be interpreted as proof of angiogenic activation rather than definitive evidence of mature, perfused vessels, and future studies should assess vessel stabilization and perfusion in parallel.

### 4.3. Promotion of Fibroblast Function and Regulation of ECM Remodeling

In diabetic wounds, dermal fibroblasts often exhibit an accelerated senescence phenotype, with markedly impaired migration, proliferation, and differentiation capacity [111]. Studies have shown that ADSC‐Exos can be efficiently internalized by fibroblasts and exert multiple protective effects under high‐glucose stress. On the one hand, ADSC‐Exos reduce oxidative stress‐related injury by decreasing the level of 8‐oxo‐2^′^‐deoxyguanosine [112]. On the other hand, preliminary evidence suggests that ADSC‐Exos may improve mitochondrial function in fibroblasts through the SMARCAL1‐Drp1‐mitochondrial dynamics pathway, thereby alleviating high glucose‐induced cellular senescence and restoring fibroblast proliferation and migration [113]. However, the general applicability of this specific mechanism and its detailed regulatory network require further validation.

ADSC‐Exos have been reported to enhance fibroblast adhesion, proliferation, and matrix production by upregulating the expression of N‐cadherin, Cyclin D1, proliferating cell nuclear antigen (PCNA), and collagen I and III in fibroblasts [90]. In addition to promoting collagen deposition and granulation tissue formation, ADSC‐Exos may also exert a certain level of regulatory control over ECM remodeling. Song et al. [114] found that ADSC‐Exos‐delivered miR‐204‐5p targets TGF‐β1 and downregulates the expression of p‐Smad2/3, collagen I, and α‐SMA. This mechanism supports cell proliferation and migration while preventing excessive myofibroblast differentiation and abnormal scar deposition. These findings suggest that ADSC‐Exos may have dual benefits in promoting tissue repair and improving scar outcomes. However, current evidence supporting the anti‐scarring effects of ADSC‐Exos remains limited. Most available studies have focused on wound closure rate and the amount of collagen deposition, whereas collagen organization, tissue mechanical properties, and long‐term scar quality have been less well evaluated. Therefore, future studies should further clarify how ADSC‐Exos regulate ECM metabolism at different stages of wound healing. Long‐term histological and functional assessments are also needed to determine whether ADSC‐Exos truly promote high‐quality wound repair.

Notably, apparently opposing regulation of TGF‐β1 has been reported in ADSC‐Exo‐based diabetic wound studies. miR‐125b‐modified ADSC‐Exos increased TGF‐β1 together with angiogenic and proliferative markers [107], whereas miR‐204‐5p carried by ADSC‐Exos suppressed TGF‐β1/Smad2/3 signaling and reduced α‐SMA and collagen I to limit excessive scarring [114]. These findings are not necessarily contradictory because TGF‐β signaling is strongly stage‐ and cell‐context‐dependent; adequate dermal TGF‐β responsiveness supports wound contraction and epithelial closure [115], whereas persistent activation can favor myofibroblast accumulation and fibrosis. Thus, future studies should define when and in which cell populations TGF‐β signaling should be enhanced or restrained.

### 4.4. Promotion of Re‐Epithelialization and Epidermal Barrier Reconstruction

ADSC‐Exos have been shown in vitro to attenuate high glucose‐induced keratinocyte injury by transferring protective signaling molecules. They can also regulate the KEAP1/NRF2/HO‐1 axis, inhibit ROS accumulation, and preserve the normal migratory and proliferative capacities of keratinocytes [92]. In addition, diabetic animal models have demonstrated that ADSC‐Exos can activate signaling pathways closely associated with epithelial repair, such as Wnt/β‐catenin signaling, thereby further enhancing keratinocyte repair activity and accelerating re‐epithelialization and granulation tissue formation. Lv et al. [116] reported that ADSC‐Exos enriched in miRNA‐21‐5p promoted local collagen deposition in diabetic wounds and improved the wound microenvironment through coordinated regulation of inflammation, epithelialization, and ECM remodeling. Moreover, multiple growth factors naturally carried by ADSC‐Exos can directly act on keratinocytes, stimulate mitotic activity, and enhance cell migration. These effects facilitate the migration of cells from the wound edge toward the wound center, leading to rapid reconstruction of the epidermal barrier and complete wound closure [117]. However, it should be emphasized that a reduction in wound area or faster wound closure does not necessarily indicate high‐quality and functional skin repair. For chronic diabetic wounds, clinically meaningful re‐epithelialization should also include restoration of the stratified epidermal structure, improved expression of tight junction and barrier‐related proteins, reconstruction of both mechanical and immune barrier functions, and partial recovery of skin appendages such as hair follicles and sweat glands. Therefore, future studies evaluating the therapeutic effects of ADSC‐Exos should not focus only on wound closure rate and superficial histological coverage. Higher‐level endpoints, including barrier function, scar quality, and functional tissue recovery, should also be incorporated.

Similarly, Wnt/β‐catenin signaling should not be interpreted as uniformly beneficial. ADSC‐derived exosomes have been reported to enhance skin‐cell proliferation and migration through Wnt/β‐catenin activation [118], yet studies of human chronic wounds showed that aberrant nuclear β‐catenin/c‐Myc signaling can inhibit keratinocyte migration and epithelialization [119]. Therefore, the therapeutic objective should be restoration of appropriately timed Wnt/β‐catenin activity rather than indiscriminate pathway activation, particularly when long‐term barrier reconstruction and scar quality are considered.

## 5. Engineering Strategies to Enhance Therapeutic Functions of ADSC‐Exos

Although native ADSC‐Exos possess multitarget therapeutic potential, their clinical application remains limited by low bioavailability, insufficient targeting ability, instability of functional cargo, and inadequate sustained efficacy in complex wounds. To overcome these limitations, researchers have developed a variety of engineering strategies, including modifying parent cells to alter exosomal contents or loading therapeutic molecules, and performing surface modifications after exosome isolation. These approaches enable the specific enrichment and efficient delivery of therapeutic proteins, miRNAs, and other noncoding RNAs and have become key strategies for further enhancing therapeutic efficacy and promoting clinical translation [120].

### 5.1. Pretreatment of Parental Cells

Hypoxic pretreatment is the most common and relatively well‐established strategy. The underlying mechanism involves mimicking the hypoxic microenvironment of injured tissues and activating HIF‐1α‐related signaling, thereby inducing ADSCs to secrete exosomes enriched with proangiogenic and anti‐inflammatory molecules. Studies have shown that the expression levels of miR‐21‐5p and miR‐100‐5p are significantly increased in hypoxia‐preconditioned ADSC‐Exos. These exosomes enhance angiogenic responses and collagen deposition in diabetic wounds by regulating pathways such as the HIF‐1α/VEGF/Akt pathway [121]. Further studies revealed that hypoxic treatment can increase the expression of circ‐Snhg11 in ADSC‐Exos. Circ‐Snhg11‐modified ADSC‐Exos enhance cell survival and maintain endothelial cell function by activating the miR‐144‐3p/NFE2L2/HIF‐1α signaling pathway, providing a new therapeutic strategy for DFUs [122]. However, standardized parameters for hypoxic pretreatment have not yet been established, and its long‐term safety and potential side effects still require comprehensive evaluation in future clinical trials.

Pharmacological pretreatments, such as small molecules, cytokines, or natural bioactive compounds are also suggested to be used to regulate the secretory profile of ADSCs, thereby enhancing the proangiogenic, anti‐inflammatory, and antioxidant functions of their exosomes [123]. Nanoparticle‐assisted conditioning may extend this concept. Silymarin/silybin‐functionalized selenium nanoparticles (SeNPs) suppressed LPS‐induced PI3K/AKT/NF‐κB activation and oxidative stress in BM‐MSCs [124]. However, that study did not analyze ADSCs or EV cargo; therefore, using SeNPs to enrich ADSC‐Exos should be regarded as a testable producer‐cell preconditioning strategy rather than an established approach. Dose, exposure time, residual nanoparticle carryover, and exosomal potency would require dedicated validation.

Genetic engineering modification is another classical strategy for obtaining functionally enhanced exosomes. Numerous studies have used lentiviral vectors, adenoviral vectors, or plasmid transfection to induce ADSCs to overexpress specific functional proteins or miRNAs. These approaches can enrich selected molecules in secreted ADSC‐Exos and improve efficacy in preclinical models, but the gain in potency is accompanied by a substantial translational burden. Transient transfection has been shown to shift EV biogenesis [125], while DNA/transfection‐reagent complexes may co‐purify with small EV preparations and confound both product characterization and attribution of biological activity [126]. Moreover, although not specific to ADSC‐Exos, a scalable engineered cell‐derived vesicle study found that only approximately half of particles carried the intended payload, illustrating that total particle number cannot be equated with the active dose [127]. Accordingly, clinical development of genetically engineered ADSC‐Exos will require well‐characterized producer cell banks, orthogonal purification, cargo‐specific release criteria, and functional potency assays rather than efficacy‐oriented engineering alone. Common ADSC pretreatment methods are summarized in Table 3.

**Table 3:** Different pretreatment strategies for ADSCs and their enhancing effects on exosomal functional properties.

Pretreatment method	Treatment condition	Wound model	Dose	Enriched miRNA/cargo	Target gene/pathway	Outcome	References
Hypoxia	O_2_ concentration <1%	DFU rat model	200 μg, intravenous injection	miR‐100‐5p	—	Significantly increased the expression of angiogenesis‐related factors VEGF and CD31, while markedly reducing the expression of inflammatory factors TNF‐α and IL‐6	[105]
Hypoxia	2% O_2_ environment	BALB/c full‐thickness defect model	100 μg, subcutaneous injection	lncRNA H19	HIF‐α	Hypoxia‐induced USP22 secretion stabilized HIF‐1 α and upregulated H19, thereby promoting skin wound healing	[128]
Hypoxia	94% N_2_, 5% CO_2_, 1% O_2_ environment	STZ‐induced C57BL/6 diabetic mouse full‐thickness skin defect model	200 μg, subcutaneous injection	miR‐144‐3p	Snhg11/NFE2L2/HIF1α	Improved survival and maintained endothelial cell function; accelerated wound healing by activating the miR‐144‐3p/NFE2L2/HIF1α signaling pathway	[122]
Drug pretreatment	50 nM empagliflozin for 48 h	db/db mouse full‐thickness skin defect model	100 μg, subcutaneous injection	—	PTEN/AKT/VEGF	Increased vascular maturity, enriched granulation tissue, and promoted more uniform and orderly collagen deposition	[129]
Drug pretreatment	100 μM metformin for 48 h	BALB/c mouse full‐thickness skin defect model	200 μL, subcutaneous injection	—	VEGFA	Enhanced the angiogenic and autophagic capacity of ADSCs, promoted angiogenesis at the wound site, and accelerated wound healing	[130]
Genetic engineering modification	E2F1 knockout	C57BL/6 mouse full‐thickness wound model	20 μL, 1 μg/μL, subcutaneous injection	miR‐130b‐5p	TGFBR3/TGF‐β pathway	Promoted fibroblast‐to‐myofibroblast differentiation, enhanced collagen deposition and tissue remodeling, and accelerated wound re‐epithelialization	[131]
Genetic engineering modification	Overexpression of mmu_circ_0000250	Diabetic rat full‐thickness skin defect model	200 μg, local injection	miR‐128‐3p	SIRT1/autophagy pathway	Promoted autophagy activation, restored EPC function, increased angiogenesis, inhibited apoptosis, and accelerated wound healing	[132]
Genetic engineering modification	Overexpression of circ‐Erbb2ip	Diabetic mouse full‐thickness skin defect model	100 μg, subcutaneous injection for 3 consecutive days	miR‐670‐5p	Nrf1/antioxidative stress pathway	Circ‐Erbb2ip enhanced wound healing in diabetic mice by increasing angiogenesis and reducing inflammatory cytokine expression	[133]
Genetic engineering modification	Overexpression of circ‐Astn1	STZ‐induced BALB/c diabetic mouse full‐thickness wound model	200 μg, subcutaneous injection	miR‐138‐5p	SIRT1/FOXO1 signaling axis	Promoted fibroblast proliferation and migration, inhibited apoptosis, and accelerated wound healing and tissue regeneration in diabetic mice	[134]
Genetic engineering modification	miR‐126‐3p transfection	SD rat full‐thickness skin defect model	0.2 mL, subcutaneous injection	miR‐126‐3p	PIK3R2	Improved wound healing rate, promoted fibroblast proliferation and migration, increased collagen deposition, and enhanced neovascularization	[135]

### 5.2. Exosome Surface Modifications

Surface functionalization involves conjugating targeting peptides, aptamers, antibodies, glycans, or adhesion molecules to the surface of the exosomal membrane to increase its binding affinity for specific cells or injured tissues. For diabetic wounds, enabling ADSC‐Exos to preferentially target macrophages, endothelial cells, or other key repair‐related cells may improve local therapeutic efficacy while reducing systemic exposure. In addition to active targeting, surface modification can also be used to enhance wound retention. For example, the introduction of collagen‐binding peptides, ECM‐adhesive molecules, or cationic modifications may increase the local adhesion of ADSC‐Exos within the exudative wound microenvironment. At present, studies directly focusing on wound‐targeted surface modifications of ADSC‐Exos remain relatively limited. However, important progress has been made in other exosome systems. Cui et al. [136] anchored a diacyl‐lipid‐modified bone‐targeting peptide onto the membrane of exosomes secreted by human induced pluripotent stem cells (iPSCs), thereby constructing a bone‐targeted engineered exosome platform with significantly enhanced osteoblast‐targeting capacity. This targeting strategy provides an important technical reference for the wound‐targeted delivery of ADSC‐Exos. By applying similar approaches, endothelial cell‐ or macrophage‐specific binding peptides could be modified onto the surface of ADSC‐Exos, potentially enabling precise enrichment of ADSC‐Exos in diabetic wounds. Notably, exogenous membrane modification may alter the conformation of native exosomal membrane proteins and affect their cellular uptake patterns, thereby influencing their biodistribution, immune recognition, and clearance pathways in vivo. Different modification densities may also induce particle aggregation or reduce biological activity. Therefore, future studies should not only focus on targeting efficiency but also evaluate the membrane integrity, cellular uptake mechanisms, biodistribution, and safety profile of modified ADSC‐Exos.

### 5.3. Loading Cargo Into Exosomes

As naturally secreted nanoscale vesicles, exosomes possess evolutionarily conserved membrane structures and intrinsic intercellular communication functions. Compared with synthetic nanocarriers, they have potential advantages in terms of biocompatibility, membrane permeability, and in vivo safety, making them natural drug delivery vehicles with both delivery efficiency and biosafety. Exogenous loading allows siRNAs, miRNAs, small‐molecule drugs, peptides, or proteins to be further encapsulated into exosomes after isolation. Common loading methods include electroporation, coincubation, sonication, extrusion, and freeze–thaw cycles, each with its own scope of application and limitations [24, 137, 138]. Electroporation is a commonly used loading method for nucleic acid therapeutics. It generates reversible micropores in the exosomal membrane through a transient electric field, allowing exogenous molecules to enter the vesicle lumen. However, excessive electric field intensity may induce exosome aggregation and membrane fusion, potentially weakening their biological activity and targeted delivery capacity. Coincubation is widely used for loading hydrophobic small‐molecule drugs because of its mild conditions and minimal damage to the membrane structure. Yan et al. [139] demonstrated that ADSC‐Exos have a high loading capacity for the active compound icariin (ICA), with an encapsulation efficiency of 92.4 ± 0.008%, which was significantly greater than that of BMSC‐Exos. These findings provide an important basis for the use of drug‐loaded ADSC‐Exos in diabetic wound therapy. Sonication increases membrane permeability through mechanical shear forces and generally achieves higher loading efficiency than coincubation and electroporation. Dox‐loaded ADSC‐Exos have demonstrated the feasibility of sonication‐based drug encapsulation, although the cited application was oncologic rather than wound‐specific and high‐intensity sonication may disrupt exosomal membrane integrity [140]. In contrast, although extrusion and freeze–thaw methods are theoretically feasible, their current applications remain relatively limited because of their low loading efficiency, the ease with which membrane stability is disrupted, and poor recovery rates. The choice of loading strategy is highly dependent on the cargo type, application scenario, and clinical translation requirements. In the complex pathological microenvironment of diabetic wounds, loading strategies should prioritize preservation of ADSC‐Exos bioactivity while balancing loading efficiency, targeting ability, and release kinetics, ultimately achieving safe, stable, and precise local delivery.

## 6. Optimization of ADSC‐Exos Delivery Strategies

### 6.1. Direct Exosome Injection Therapy

Local injection of ADSC‐Exos into the wound margin or wound bed is the most direct administration route, which is relatively simple to perform and enables rapid local enrichment of ADSC‐Exos. In preclinical studies, local injection has shown significant pro‐healing effects in multiple diabetic animal wound models. Studies have shown that, in a diabetic rat DFU model, compared with noninjection, local injection of miR‐125b‐modified ADSC‐Exos significantly increased the expression of CD34, Ki‐67, VEGF, and TGF‐β1 in wound tissue; reduced inflammatory infiltration; and markedly improved granulation tissue formation and wound healing [141]. Similarly, ADSC‐Exos with high miR‐146a‐5p expression effectively regulate the JAZF1‐mediated angiogenic pathway after local injection, thereby accelerating diabetic wound healing [104]. To date, clinical evidence specifically supporting ADSC‐derived EV therapy for diabetic wound treatment remains limited, and most available studies are still restricted to preclinical investigations [142]. However, the core limitation of this strategy lies in the mismatch between pharmacokinetics and the pathological course of chronic wounds. After injection, ADSC‐Exos have a relatively short retention time at the wound site and are easily washed away by tissue fluid or degraded enzymatically. A single injection is therefore insufficient to maintain a sustained therapeutic concentration, whereas repeated injections may increase infection risk and patient discomfort. Thus, injection therapy may be more suitable for small‐area or acute‐phase wounds, or as an initiating approach in combination therapy, rather than as a long‐term strategy for chronic nonhealing wounds.

### 6.2. Hydrogel Delivery Systems

Construction of biomaterial‐based delivery systems adapted to the pathological characteristics of wounds has become a key approach for improving the retention, stability, and bioavailability of ADSC‐Exos. ECM‐mimetic biomaterials, which combine biocompatibility, structural biomimicry, and sustained‐release capacity, represent preferred platforms for ADSC‐Exos delivery. Song et al. [73] developed an ADSC‐Exos bioreinforced ECM hydrogel using natural ECM components secreted by ADSCs and anchored ADSC‐Exos within it. This not only achieved long‐term release of ADSC‐Exos, but also preserved the bioactive signals of the ECM, thereby synergistically accelerating diabetic wound healing and skin regeneration. These materials exhibit excellent biocompatibility and can effectively fill irregular wounds, addressing the limitations of conventional dressings, such as poor conformability and insufficient local drug retention. Collagen, a major component of the ECM, also provides an ideal three‐dimensional scaffold for ADSC‐Exos delivery. Fereshteh et al. [143] constructed a collagen‐based three‐dimensional porous scaffold encapsulating ADSC‐Exos, which mimics the natural ECM structure and provides a favorable microenvironment for the infiltration, migration, and proliferation of repair‐related cells, such as fibroblasts and endothelial cells, thereby further amplifying the reparative effects of ADSC‐Exos.

The three‐dimensional hydrophilic network of hydrogels not only provides physical protection for ADSC‐Exos, but also enables controllable, sustained release through the coordinated effects of swelling, degradation, and diffusion, thereby effectively prolonging their residence time and therapeutic action at the wound site. After ADSC‐Exos are loaded into a chitosan (CS) and polyethylene glycol (PEG) composite hydrogel, the exosomes are continuously released as the material degrades, which significantly enhances endothelial cell migration, reduces oxidative stress, and promotes angiogenesis in vitro [144]. Similarly, He et al. [145] developed a bioactive phosphorylcholine hydrogel based on gelatin methacryloyl and methacrylated alanine via free‐radical polymerization. This bioactive hydrogel binds ADSC‐Exos through specific phosphorylcholine interactions, ensuring their stability and sustained release. Moreover, antibacterial metal organic frameworks, which are activated by blue light and used to improve the bioavailability of curcumin, are physically incorporated into the hydrogel matrix. Together, these components synergistically promote chronic wound healing and complete skin regeneration, resulting in superior wound‐healing effects compared with ADSC‐Exos alone.

In addition to synthetic polymeric materials, natural biomaterials have also attracted extensive attention because of their superior bioactivity. One study developed a bilayer patch based on porcine pericardium‐derived decellularized extracellular matrix (dECM) for loading ADSC‐Exos. This patch not only preserves the natural three‐dimensional architecture and bioactive components of the ECM, but also enables local enrichment and sustained release of ADSC‐Exos [146].

To address the pathological features of diabetic wounds, including excessive ROS, persistent inflammation, weak electrical conductivity, and irregular morphology, researchers have further designed multifunctional hydrogel systems with environmental responsiveness and functional adaptability. By incorporating polyphenolic compounds or intrinsically antioxidative polymers, antioxidant hydrogels can scavenge excess ROS at the wound site while sustainably releasing ADSC‐Exos, thereby reducing oxidative stress‐induced damage and preserving exosomal bioactivity [147]. Beyond serving as depots for native ADSC‐Exos, hydrogels may also localize drug‐loaded exosome formulations. In a rat psoriasis model, EGCG nanoparticle‐loaded exosomes showed greater anti‐inflammatory and anti‐apoptotic effects than exosomes or EGCG nanoparticles alone [148]. Although this evidence is not derived from diabetic wounds or ADSC‐Exos, it provides a proof‐of‐concept for combining exosomal small‐molecule delivery with hydrogel‐mediated local retention. Conductive hydrogels integrate electrically conductive components to mimic the physiological transmission of skin bioelectrical signals, thereby promoting nerve reinnervation and wound contraction and improving the quality of full‐thickness skin regeneration [149–151]. In addition, self‐healing injectable hydrogels constructed through dynamic chemical bonds, such as Schiff base linkages and host–guest interactions, can fill wounds in a minimally invasive manner, form in situ, and adapt to dynamic wound deformation, greatly enhancing clinical convenience and wound coverage performance [152–154].

Electrospun nanofibrous dressings represent another promising vehicle for ADSC‐Exos delivery. Their highly porous structure and large specific surface area can mimic the fibrous network of the natural ECM, while also providing the conventional functions of wound dressings, including physical protection, gas exchange, and maintenance of a moist wound environment [155]. These membrane‐based materials also serve as physical barriers against bacterial invasion while permitting gas exchange. Their combination with ADSC‐Exos may therefore offer a novel therapeutic approach for diabetic wound treatment.

Nanomaterials can be engineered to combine controlled release with antimicrobial, antioxidant, immunomodulatory, and microenvironment‐responsive functions, thereby addressing several interacting barriers to chronic wound repair simultaneously [156]. Magnetic nanoparticles provide a more specific example of this modular approach. Magnetite nanoparticles can be prepared by co‐precipitation, and the choice of precipitating base measurably alters their physicochemical and biological properties [157], underscoring the need to control synthesis parameters before integration with biologic carriers. Separately, iron oxide‐loaded MSC‐derived exosomes have shown magnetic‐field‐enhanced accumulation and cutaneous wound repair [158]. Together, these findings support exploration of magnetically responsive ADSC‐Exo hybrid carriers for diabetic wounds, but do not yet establish efficacy. A related hybrid concept is supported by an inflammation model in which AD‐MSC‐conditioned medium combined with mono‐ or dual‐doped TiO_2_ nanoparticles produced anti‐inflammatory and antioxidant effects, with Cu/Zn‐doped TiO_2_ showing the strongest response [159]. Because the conditioned medium contains both soluble factors and EVs and is not equivalent to purified ADSC‐Exos, these findings do not establish an ADSC‐Exo/TiO_2_ therapy; rather, they support further testing of secretome‐ or exosome‐inorganic nanoparticle hybrids, with direct evaluation of particle–vesicle interactions, oxidative balance, cytotoxicity, and wound‐specific efficacy. Engineered exosomes represent a biologically derived nanotherapeutic within this spectrum, but their endogenous communication capacity must be balanced against heterogeneity in cargo loading, biodistribution, manufacturing reproducibility, and scale‐up requirements [160]. Programmable nanostructures such as tetrahedral framework nucleic acids further illustrate how nanoscale architecture can both deliver therapeutic cargo and modulate cellular behavior; however, evidence specific to diabetic wounds remains predominantly preclinical, and long‐term degradation, clearance, dose standardization, and safety require further validation [161]. Thus, nanomedicine is more appropriately viewed as a modular strategy that may complement exosome therapy, with platform selection guided by mechanism, wound phenotype, and translational feasibility rather than by nanoscale formulation alone.

However, material‐based delivery systems still face several critical challenges in clinical translation. Key issues requiring systematic validation and optimization include the biocompatibility and safety of material degradation products, the release kinetics of ADSC‐Exos, the effects of sterilization processes on exosomal bioactivity, and the adhesive performance and structural stability of materials in the highly inflammatory and exudative wound microenvironment. Accordingly, future studies should establish a comprehensive evaluation framework covering multiple dimensions, including the physicochemical properties of the materials, the release kinetics of exosomes, the efficiency of bioactivity preservation, and the histological quality of tissue repair. Such efforts will help promote the translation and development of ADSC‐Exos biomaterial composite systems from basic research to clinical application.

### 6.3. Integration of 3D Printing Technology for Exosome Delivery

The integration of exosomes with 3D printing technology has emerged as a promising direction in tissue engineering and regenerative medicine. 3D‐printed scaffolds offer several advantages, including customizable geometric structures, multimaterial compatibility, adjustable porosity, and the ability to fit irregular tissue defects, making them theoretically ideal for treating complex, deep, and heterogeneous diabetic wounds. While research on 3D‐printed exosome‐loaded wound dressings for ADSC‐Exos is still in the exploratory phase, successful applications of other types of exosomes in 3D‐printed scaffolds provide valuable insights for this field. For example, Dutta et al. [162] developed a bioink composed of collagen and decellularized extracellular matrix (COL@d‐ECM). This bioink not only offers excellent mechanical properties and biocompatibility, but also allows stable loading of M2 macrophage‐derived exosomes, which significantly accelerate wound healing through immune modulation. Additionally, Luo et al. [163] applied a dual‐biomimetic strategy by combining 3D‐printed titanium alloy scaffolds with hydrogel microspheres loaded with hypoxia‐induced exosomes. This approach enables the long‐term, controlled release of exosomes and promotes angiogenesis and tissue regeneration during bone defect repair. These studies suggest that 3D printing technology has the potential to provide an innovative and tailored approach for ADSC‐Exos delivery in the treatment of diabetic wounds, particularly for enhancing the controlled release and localized therapeutic effects of exosomes within the complex wound microenvironment.

From the perspective of practical application, the design of ADSC‐Exos delivery systems should match the pathological characteristics of diabetic wounds. Direct injection is simple to perform, but its local retention time is limited, and the effective utilization of exosomes is easily reduced by exudate washout and interstitial diffusion. Hydrogel systems possess good wound conformity, moisture retention, and design flexibility, making them especially suitable for wounds with high inflammation, elevated ROS levels, and irregular morphology. They are therefore considered one of the most promising local delivery platforms at present. Electrospun nanofibers can mimic the fibrous network of the natural ECM while also providing structural support and barrier protection, making them more suitable for superficial wounds or wounds requiring long‐term coverage. In contrast, 3D‐printed scaffolds offer unique advantages in terms of geometric customization, multimaterial integration, and functional partitioned delivery, and are theoretically well suited for deep or complex irregular defects. However, their application in diabetic wounds is still at an early exploratory stage. Therefore, no single delivery platform has absolute superiority. A more rational research direction is to design delivery systems according to specific clinical scenarios, including wound depth, exudate level, infection risk, anatomical location, and the presence or absence of tissue defects. In addition, the release behavior of ADSC‐Exos should match the different stages of wound healing to achieve more precise local therapy.

## 7. Clinical Translation Prospects and Challenges

Although ADSC‐Exos have shown promising wound‐healing potential in multiple diabetic wound animal models, most existing studies remain at the preclinical stage. Searches of international clinical trial registries and ClinicalTrials.gov data indicate that human clinical studies directly targeting ADSC‐Exos for diabetic wounds or DFUs are still quite limited. Some MSC‐derived exosome trials have entered the safety and dose‐exploration stages, but publicly available efficacy data remain insufficient, so no definitive conclusions regarding clinical effectiveness can yet be drawn. Therefore, further clinical translation of ADSC‐Exos for diabetic wound treatment urgently requires more high‐quality clinical evidence.

The lack of clinical evidence for the use of ADSC‐Exos in the treatment of diabetic wounds is largely attributed to several translational bottlenecks in various areas, such as source variability, production, quality control, safety, and clinical evaluation. First, donor heterogeneity is a significant factor influencing product stability. Variables such as age, sex, metabolic status, underlying disease, medication history, and fat tissue collection site can all alter the secretion profile and exosomal cargo composition of ADSCs. This is especially true for autologous ADSCs from diabetic patients, whose functionality may be compromised due to prolonged hyperglycemia, chronic inflammation, and metabolic dysregulation [164]. Importantly, whether this cellular dysfunction is directly inherited by autologous ADSC‐Exos remains unresolved. Mechanistically, T2D/obesity‐derived human ADSCs show enhanced glycolytic/inflammasome activity and reduced immunoregulatory function, providing a plausible upstream basis for altered exosomal cargo sorting [165]. Although obesity is not equivalent to diabetes, direct EV evidence from metabolically compromised human ADSCs supports this concern: obesity altered the miRNA cargo of ADSC‐derived EVs and reduced their ability to suppress NF‐κB‐associated inflammation and apoptosis in recipient cells [166]. However, adipose‐derived MSCs from patients with diabetic kidney disease exhibited extensive mRNA/miRNA remodeling, yet their conditioned medium retained angiogenic activity comparable to that of controls [167]. Thus, diabetes‐related donor effects may represent context‐dependent qualitative cargo reprogramming rather than a uniform loss of potency.

Second, large‐scale standardized production and quality control systems for ADSC‐Exos are still under development. A GMP‐oriented control strategy should begin upstream rather than rely only on final‐product testing. Because fetal bovine serum (FBS) and other human‐ or animal‐derived supplements can introduce exogenous vesicles, protein impurities, lot variability, and infectious‐agent risks, chemically defined, xeno‐free or animal‐component‐free media and recombinant reagents should be prioritized whenever technically feasible. Studies using serum‐/xeno‐free or GMP‐grade media indicate that such conditions can maintain core MSC‐EV characteristics and biological activity, although comparability must be re‐established for each ADSC manufacturing platform [168, 169]. Viral safety requires particular caution. EVs and enveloped viruses overlap substantially in size and physicochemical behavior, making conventional virus‐removal or inactivation steps difficult to transfer directly from conventional biologics without simultaneously depleting or damaging EVs [170].

Third, EV quality should be managed using a quality‐by‐design framework that links critical quality attributes (CQAs) to critical process parameters (CPPs) and in‐process controls rather than relying primarily on end‐product particle size and CD9/CD63/CD81 measurements. Acceptable ranges of candidate CPPs should be established experimentally according to their effects on clinically relevant CQAs, including identity, particle concentration and size distribution, purity, cargo or functional potency, microbiological safety, and stability, with formal comparability assessment after scale‐up or major process changes [171]. In parallel, the distribution, metabolism, clearance, optimal dosing, and local retention of ADSC‐Exos remain incompletely defined, and potential off‐target effects, abnormal angiogenesis, immune interference, and risks introduced by engineered modifications require systematic evaluation.

Finally, the regulatory strategy should be defined early and on a jurisdiction‐specific basis rather than assuming a single product category. Native ADSC‐Exos, genetically engineered EVs, and EV‐biomaterial combination products may require different chemistry, manufacturing and control packages; therefore, product definition, mechanism‐linked potency assays, release specifications, comparability plans, and post‐treatment safety monitoring should be aligned with the intended product design and clinical use [25, 171]. Overall, clinical translation will depend not only on demonstrating biological efficacy but also on establishing a reproducible manufacturing process in which raw materials, CPPs, CQAs, viral safety, and release criteria are prospectively controlled.

**Figure 3:**
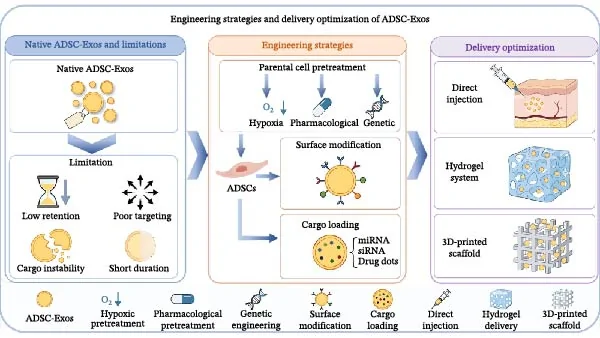
Engineering strategies and delivery optimization of ADSC‐Exos. Native ADSC‐Exos are limited by low retention, poor targeting, cargo instability, and short action. Parental cell pretreatment, surface modification, and cargo loading enhance exosome function, while hydrogels and 3D‐printed scaffolds improve retention and sustained release, optimizing therapeutic efficacy.

In parallel, the development of advanced delivery platforms, particularly 3D printing technologies and smart responsive hydrogels, may allow ADSC‐Exos to be released in a controlled manner according to changes in the wound microenvironment, which could further increase their bioavailability (Figure 3). Engineering and biomaterial‐based delivery may improve local retention and controlled release in preclinical models, but these gains have not yet been established in humans. Human pharmacokinetics, biodistribution, repeated‐dose safety, and reproducible release profiles remain insufficiently characterized [172]. At present, compared with systemic administration, local delivery combined with biomaterial‐based retention systems may represent a more practical near‐term translational strategy, as this approach can minimize systemic exposure while increasing the effective local concentration at the wound site. Importantly, several clinical trials based on mesenchymal stem cell‐derived exosomes have already been registered worldwide, including some phase I/II studies listed on ClinicalTrials.gov, with current efforts focused mainly on safety evaluation and dose exploration [173, 174]. Future clinical studies should be conducted in carefully selected patient populations, with standardized endpoints and adequate follow‐up. In addition to wound closure, these studies should also assess recurrence, tissue quality, and safety outcomes. Accordingly, ADSC‐Exos should currently be regarded as a translational candidate supported mainly by preclinical evidence rather than as a clinically established therapy; progression will depend on resolving manufacturing, potency, regulatory, and long‐term safety uncertainties [175].

## 8. Conclusion

The impaired healing of chronic diabetic wounds is a complex pathological process driven by multiple interconnected mechanisms, including persistent inflammatory activation, elevated oxidative stress, impaired angiogenesis, aberrant ECM remodeling, and defective re‐epithelialization. As a cell‐free therapeutic strategy characterized by diverse molecular cargo and relatively low immunogenicity, ADSC‐Exos may deliver miRNAs, proteins, and other bioactive molecules to synergistically regulate inflammatory responses, promote immune remodeling, increase angiogenesis, improve fibroblast function, and facilitate keratinocyte repair, thereby exerting a systemic modulatory effect on the diabetic wound microenvironment. In recent years, advances in engineered exosomes, ADSC preconditioning, and intelligent delivery materials have further improved the targeting ability, retention, and therapeutic persistence of ADSC‐Exos, providing new technical support for their clinical translation.

Nevertheless, the clinical application of ADSC‐Exos still faces substantial challenges. Heterogeneity in donor sources, inconsistency in preparation processes and quality control standards, the lack of robust potency evaluation systems, unclear biodistribution and metabolic behavior in vivo, insufficient evidence regarding long‐term safety, and an undefined regulatory pathway all continue to hinder further development. Future studies should focus on the systematic elucidation of key effector molecules and their signaling networks, while also addressing donor selection criteria, GMP‐compliant manufacturing processes, indication‐oriented quality control systems, locally targeted delivery strategies, and well‐designed high‐quality clinical trials. Coordinated progress in mechanistic investigation, process standardization, potency assessment, and clinical validation will therefore be required to determine whether ADSC‐Exos can achieve a reproducible and acceptable benefit‐risk profile in chronic diabetic wounds.

## Author Contributions

Hongtao Wang conceived the review and revised the manuscript. Danna Yao drafted the manuscript. Yujie Xiao, Mali Zhou, and Panpan Sun contributed to the literature collection and editing of the text and tables. Ling Wang contributed to critical revision of the manuscript.

## Funding

This work was supported by the Social Development Foundation of Shaanxi Province (Grant 2023‐ZDLSF‐37).

## Disclosure

All the authors read and approved the final manuscript.

## Ethics Statement

The authors have nothing to report.

## Consent

The authors have nothing to report.

## Conflicts of Interest

The authors declare no conflicts of interest.

## Data Availability

Data sharing is not applicable to this article as no datasets were generated or analyzed during the current study.
